# The juvenile gangliosidoses: A timeline of clinical change

**DOI:** 10.1016/j.ymgmr.2020.100676

**Published:** 2020-11-14

**Authors:** Kelly E. King, Sarah Kim, Chester B. Whitley, Jeanine R. Jarnes-Utz

**Affiliations:** a Department of Pediatrics, University of Minnesota, 2450 Riverside Avenue, Minneapolis, MN 55454-1450, USA; b Gene Therapy Center, University of Minnesota, 420 Delaware St SE, MMC 391, Minneapolis, MN 55455-0341, USA; c Department of Experimental and Clinical Pharmacology, College of Pharmacy, University of Minnesota, 420 Delaware St SE, MMC 446, Minneapolis, MN 55455-0341, USA; d Advanced Therapies Program, University of Minnesota (UMMC) and Fairview Hospitals, Minneapolis, MN 55454, USA

**Keywords:** GM1-gangliosidosis, Tay-Sachs disease, Disease progression, Child, Adolescent, Inherited metabolic diseases

## Abstract

**Background:**

The gangliosidoses are rare inherited diseases that result in pathologic accumulation of gangliosides in the central nervous system and other tissues, leading to severe and progressive neurological impairment and early death in the childhood forms. No treatments are currently approved for the gangliosidoses, and development of treatments is impaired by limited understanding of the natural history of these diseases.

**Objective:**

The objective of this study is to improve understanding of the juvenile gangliosidoses phenotypes and the late-infantile phenotypic subtype.

**Methods:**

Through a prospective natural history study of subjects with juvenile GM1- and GM2-gangliosidosis, a timeline of clinical changes was developed for the classic juvenile phenotypes and the late-infantile phenotypes and results of serial neurodevelopmental testing was analyzed.

**Results:**

Several candidate ‘outcome measures’ were identified: changes in ambulation and verbalization skills, the communication domain from neurodevelopmental testing and the caregiver-reported socialization domain.

**Conclusions:**

The most common symptoms leading caregivers to seek a genetic diagnosis were changes in ambulation and verbalization.

## 1 Introduction

The gangliosidoses are rare, autosomal recessive diseases caused by specific lysosomal enzyme deficiencies that result in pathologic accumulation of gangliosides in the central nervous system and other tissues eventually leading to severe and progressive neurodegeneration [[1], [2], [3]]. In GM1-gangliosidosis, mutations in the *GLB1* gene result in defective production of β-galactosidase-1 and accumulation of GM1-gangliosides [2,3]. In GM2-gangliosidoses (Tay-Sachs disease and Sandhoff disease), mutations causing defects in β-hexosaminidase A activity lead to the accumulation of GM2-gangliosides. The β-hexosaminidase A enzyme is a heterodimer composed of α- and β-subunits, both of which are necessary for its activity [4]. In Tay-Sachs disease, mutations in the *HEXA* gene result in a defective α-subunit [4], whereas in Sandhoff disease, mutations in the *HEXB* gene prevent normal β-subunit production [[1], [2], [3]].

Phenotypes of GM1- and GM2-gangliosidoses can be divided into three types, determined by the age of onset of prominent clinical symptoms: infantile, juvenile, and late-onset (adult onset). In the infantile forms of both GM1-and GM2-gangliosidoses, symptoms become apparent by 6 months of age, and death occurs in early childhood, most often between 2.5 and 4.5 years of age [1,5,6]. The pattern of clinical changes and timelines in which they occur in the rapidly progressive infantile phenotypes of both GM1- and GM2-gangliosidosis are markedly similar and have been described previously [1,5,6].

Characterization of the juvenile gangliosidosis phenotypes has been largely provided through single patient case reports and case studies [4,7,8]. Among the available case reports, dysarthria and ambulation difficulties are frequently described as common presenting symptoms [9,10].

Differing views about the common age of symptom onset for the juvenile gangliosidosis phenotypes have been presented [1,11]. Regier et al. describe an age of symptom onset of 2 to 10 years for the more commonly recognized classic juvenile GM1-gangliosidosis (also referred to as type II juvenile), overlapping with an age of 1 to 3 years for the late-infantile phenotype subset (also referred to as type II late-infantile) [10]. In contrast, Tuteja, et al. describe a late-infantile form of GM1-gangliosidosis with symptom onset between 6 months and to 36 months, and an adult form with onset between 3 and 30 years [12].

Lifespans vary considerably for patients with juvenile gangliosidoses, with reports ranging from mid-childhood to early adulthood [1,2,7]. In the late-infantile subtype of juvenile GM1-gangliosidosis, lifespan may extend from mid-childhood to later childhood, but is not anticipated to reach adolescence [1]. This is in contrast to the infantile forms of GM1- and GM2-gangliosidosis, in which death occurs quite consistently during a relatively narrow window of time ranging from 2.5 to 4.5 years [1,5,6].

The existence of a late-infantile phenotype of GM1-gangliosidosis, as a subtype of the juvenile phenotype, is acknowledged broadly and described in the literature [1,9]. However, a late-infantile GM2-gangliosidosis has not been formally recognized and, to date, has been described only in a few case reports [13,14]. A single case report of late-infantile Tay-Sachs disease by Eiris et al., reported that this subject died at 5 years 8 months of age [13].

There are currently no FDA-approved treatments for gangliosidosis diseases, but a number of treatments are being investigated, including gene therapies, chaperone therapies, and forms of recombinant enzyme replacement therapy [1].

Clinical trial design for juvenile gangliosidoses has been hampered by the difficulty in establishing more homogeneous cohorts for study. This is due not only to extremely small sample sizes and heterogeneity within phenotypes, but also by inconsistency and confusion related to varying classification systems. The very nature of a rare, autosomal recessive disease, such as the gangliosidoses, in which there is a spectrum of disease severity and for which many different allele mutations are causative, precludes having a truly homogenous phenotype for clinical trial cohorts. Nevertheless, there remains a need to be able to more clearly distinguish disease phenotypes and sub-phenotypes of the gangliosidoses, with ultimate goals being to identify clinical trial outcomes that are appropriate and meaningful for the respective phenotype, allow appropriate selection of children for clinical research trial cohorts, as well as to aid in clarifying prognostic expectations for families and to improve consistency of communication between clinicians.

The objective of this study is to improve understanding of the juvenile gangliosidoses and the phenotypic subtype known as “late-infantile gangliosidosis” through development of a timeline of clinical changes experienced by patients and analysis of serial neurodevelopmental testing. The authors hypothesize that development of a timeline of clinical changes that occur in the juvenile gangliosidoses would more clearly distinguish and characterize the late-infantile phenotype and classic juvenile phenotypes. Furthermore, the authors hypothesize that developing a timeline of clinical changes would not only show that the late-infantile phenotype, as its name implies, would consistently have initial onset of symptoms during the late-infantile period of life, or very close to or around the age of 12 months, and thus would be distinguished by an earlier onset of symptoms, but also would consistently have a more aggressive and rapidly progressive disease progression, when compared to the classic or traditional juvenile phenotype.

## 2 Subjects and methods

### 2.1 Subjects

Subjects participating in this analysis were enrolled in the Natural History of Gangliosidoses Study at the University of Minnesota (clinicaltrials.gov, NCT00668187 and NCT02030015) of the Lysosomal Disease Network (U54NS0657698). This study was also part of the National Institutes of Health Rare Diseases Clinical Research Network. The study protocol was approved by the University of Minnesota institutional review board, and written informed consent was obtained from the parents or legal guardians of each child before study entry.

Subjects were included in this analysis if they had a diagnosis of juvenile or late-infantile GM1- or GM2-gangliosidosis. The diagnosis was confirmed biochemically by enzyme assays and, whenever possible, by sequencing of *GLB1* gene (GM1-gangliosidosis), *HEXA* gene (Tay-Sachs disease), or *HEXB* gene (Sandhoff disease). No exclusion criteria were applied. Subjects were initially designated as late-infantile phenotype, as its name implies, if the subject's initial onset of symptoms leading caregivers to seek a diagnosis, occurred during the late-infantile period of life, specifically, in the latter half of the first year of life or close to the age of 12 months. Subjects were classified as having the traditional, or classic, juvenile phenotype if the initial onset of symptoms leading caregivers to seek a diagnosis occurred after the infantile period of life (i.e, after the first year of life). Medical history of each subject was obtained and then subjects were followed prospectively and new clinical changes were documented in ongoing fashion.

### 2.2 Clinical evaluations

Clinical changes were identified prospectively during ongoing standard clinical care and retrospectively through review of medical records and interviews with caregivers. Comprehensive clinical evaluations were performed by specialists in lysosomal diseases, as part of standard clinical care at the University of Minnesota. Specialists included a geneticist, neurologist, cardiologist, pharmacotherapist, neuropsychologist, ophthalmologist, and pulmonologist. Ability for subjects to be evaluated by certain specialists depended on the participant's subject's medical insurance coverage. In addition, all subjects were followed by healthcare providers near their homes, including a primary care physician and a neurologist.

Relevant clinical features were recorded, including age at the time of observation of the first clinical symptom that prompted the caregivers to seek a diagnosis, age of onset and/or identification of other symptoms, and neurodevelopmental test results, when available. Follow-up visits and evaluations were done once yearly whenever possible, and more frequently in some subjects, and were subject to healthcare coverage of each subject. Follow-up phone calls to caregivers were attempted when visits to clinic were not possible.

A timeline of clinical change was documented for each subject. Distinguishing features and similarities between the GM1- and GM2-gangliosidosis groups were noted.

Magnetic resonance imaging exams were performed to evaluate brain changes and to measure changes in cerebral structure volumes. These results have been previously reported by Nestrasil, et al. [15].

### 2.3 Neurodevelopmental evaluations

Neurodevelopmental evaluations were done as part of clinical care, depending on the minimum allowance by subjects' healthcare plans. All neurodevelopmental evaluations were done at the University of Minnesota. For each subject, the neuropsychologist chose the most appropriate neurodevelopmental test(s) to be administered. The Bayley Scales of Infant and Toddler Development®, Third Edition (Bayley-III) is a validated evaluation of early cognitive, language, and motor skills for children from 1 to 42 months, 15 days of age [16]. It was administered directly to patients by the neuropsychologist, including to children older than 42 months, 15 days of age when measures standardized for older children were not appropriate for their level of functioning. The test takes between 30 and 90 min to complete. Age-equivalents and standard scores (with a mean of 100 and a standard deviation of 15) are generated from a normative population of children 42 months, 15 days of age or less. For those over 42 months, 15 days, only age-equivalents are derived. Other cognitive tests, such as the Kaufman Assessment Battery for Children, Second Edition (KABC-II), or the Mullen Scales of Early Learning (Mullen) were administered when appropriate for the subjects' age, their ability level, and their needs for clinical care [17,18]. For instance, the KABC-II could be administered instead of the Bayley-III if the child was over 42 months old and functioning at a level of a child 36 months of age or greater.

To quantify functioning of children in daily life, parents or caregivers completed the Vineland Adaptive Behavior Scales™, Second Edition (Vineland-II) or Third Edition (Vineland-III), during a semi-structured interview of the caregivers with the neuropsychologist at the University of Minnesota [19,20]. This measure is standardized for individuals from birth to 90 years of age and evaluates 4 domains of skills (Functional Communication, Daily Living Skills, Social Skills, and Motor Skills) and 11 subdomains. Derived from a normative population, the test provides standard scores (with a mean of 100 and a standard deviation of 15) for each domain as well as an overall ability score (Adaptive Behavior Composite score) and age-equivalent scores for each subdomain.

### 2.4 Statistical analysis

Clinical changes and medical interventions were reported for each subject. The risk of clinical changes and medical interventions was estimated by Kaplan-Meier analysis (Prism 6, GraphPad). If the information was missing, data were censored at the last evaluation or patient death. The Kaplan-Meier curves were used to calculate the median age of clinical changes or medical interventions.

## 3 Results

### 3.1 Patient demographic characteristics

A total of 19 subjects were enrolled in this natural history analysis. Thirteen subjects had a diagnosis of GM1-gangliosidosis: eight with classic juvenile GM1-gangliosidosis (jGM1) and five with late-infantile GM1-gangliosidosis (liGM1) (Table 1). Six subjects had a diagnosis of GM2-gangliosidosis: three with classic juvenile Tay-Sachs disease (jTS) and three with late-infantile Tay-Sachs disease (liTS). Twelve subjects were female (63%), including all six GM2-gangliosidosis subjects. Most subjects were living in the United States and most were of Caucasian or European origin. Two subjects with jGM1 were siblings (subjects #7 and #8) and one patient with liTS had consanguineous parents (subject #19). Notably, only one subject with juvenile Sandhoff disease was enrolled, and there were insufficient data to include from this individual.

**Table 1:** Demographic information and diagnosis of patients.

	Patient characteristics			Diagnostic criteria			
Patient number	Diagnosis	Gender	Race (per medical record)	Genotype	Age at clinical findings (months)	Initial clinical findings leading to diagnosis	Age at diagnosis (months)
#1	jGM1	F	Korean, German, Irish	c.464 T > G (p.Leu155Arg) / unknown	48	Falling frequently off her chair, followed by noticeable gait abnormalities and subtle abnormalities in tongue movements	65
#2	jGM1	F	Irish, German, Korean	c.442C > A (p.Arg148Ser) / c.574 T > C (p.Tyr192His)	30	Falling frequently without apparent reason, followed by dyspraxia, speech delay, general developmental delays, demyelination on brain MRI, inattention	125
#3	jGM1	F	Caucasian	c.602G > A (p.Arg201His) / c.1051C > T (p.Arg351Ter)	48	Started losing words	144
#4	jGM1	M	Caucasian	c.602G > A (p.Arg201His) in exon 6 / c.902C > T (p.Ala301Val) in exon 8	24	Developmental delays in speech	96
#5	jGM1	F	Chinese	c.245C > T (p.Thr82Met) / c.367G > A (p.Gly123Arg)	18	Gait abnormalities, difficulties with balance	48
#6	jGM1	M	Caucasian	Unavailable	54	Clumsiness, frequent falls, difficulties with speech and new onset of stuttering	96
#7	jGM1	M	Caucasian	Homozygous c.602C > T (p.Arg201Cys)	24	Esotropia, hyperopia, astigmatism, amblyopia	66
#8	jGM1	M	Caucasian	Homozygous c.602C > T (p.Arg201Cys)	24	Speech delays	19
#9	liGM1	M	Caucasian	c.602C > T (p.Arg201Cys)/ c.1733A > G (p.Lys578Arg)	20	Abnormal gait, hypotonia	22
#10	liGM1	M	French Canadian, Irish, Polish	c.75 + 2dupT (IVS1 + 2dupT) / c.1667 T > C (p.Phe556Ser)	17	Difficulties with speech and balance, began having frequent falls	20
#11	liGM1	M	Canadian	c.601C > T (p.Arg201Cys) / c.1667 T > C (p. Phe556Ser)	18	Balance difficulties and loss of words	35
#12	liGM1	F	Filipina	c.683 T > C (p.Leu228Pro) / c.2006dupA (p.Asn669Lysfs*53)	18	Delays in walking and speech development	39
#13	liGM1	F	Caucasian	p.Arg201Cys / p.LEU463Pro	13	Difficulty with balance during walking	34
#14	jTS	F	Mexican, Italian, German, Native American, Dutch	c.1274_1277dupTATC / p.Gly461Val	36	Difficulties with balance, abnormal gait, frequent falling, onset of stuttering	75
#15	jTS	F	Caucasian	c.77G > A (p. Trp26STer) / c.1496G > A (p.Arg499His)	60	Stuttering, difficulties in finding words	96
#16	jTS	F	Egyptian, Italian, Lithuanian	p.Arg504His (CGC > CAC) (c.1511G > A in exon 13)	36	Difficulties with balance, frequent falling	86
#17	liTS	F	Irish, Norwegian, German	c.570G > T (splice mut.) / c.1273_1277delATATCinsATATCTATC (p.Tyr427IlefsTer5)	14	Difficulty ambulating and reduced interest in social interactions	22
#18	liTS	F	French Canadian	c.533G > A (p.Arg178His) / c.1495C > T (p.Arg499Cys)	20	Never learned to walk	36
#19	liTS	F	South Indian	Homozygous for exon 7, c.745C > T (p.Arg252Cys)	16	Difficulties with furniture surfing, losing words	22

jGM1, juvenile GM1-gangliosidosis; jTS, juvenile Tay-Sachs disease; liGM1, late-infantile GM1-gangliosidosis; liTS, late-infantile Tay-Sachs disease.

### 3.2 Symptom onset and age of diagnosis

In the classic juvenile forms, age of first noted symptom that ultimately led to a diagnosis ranged between 18 and 54 months in subjects with jGM1, with all except one subject having their first noted symptom at age 24 months or later, and between 36 and 60 months for subjects with classic juvenile GM2-gangliosidoses (i.e., jTS). In subjects with late-infantile disease, the age of first noted symptom leading to diagnosis ranged from 13 to 20 months in subjects with liGM1 and 14 to 20 months in subjects with liTS. The median age at diagnosis was 81 months for jGM1 and 18 months for liGM1; it was 36 months for jTS and 22 months for liTS patients. Enzyme assay was performed in all subjects as part of diagnostic testing. Mutation analysis results of respective GLB1, HEXA or HEXB gene were available from the medical record for all, except 1 subject.

### 3.3 Clinical changes

#### 3.3.1 Ambulation and verbalization developmental milestones

All subjects with classic juvenile disease and late-infantile disease met their first-year developmental milestones at normal developmental time points (Supplementary Tables 1 and 2). All subjects with jGM1 or jTS learned to walk independently at 9–14 months of age (Supplementary Table 1). Two of five subjects with liGM1 and all three subjects with liTS never learned to walk independently, but they were able to crawl or take steps with assistance. Similarly, all subjects with classic juvenile disease and all subjects with late-infantile disease learned to form words before 13 months of age, and in most cases, to form sentences.

**Fig. 1:**
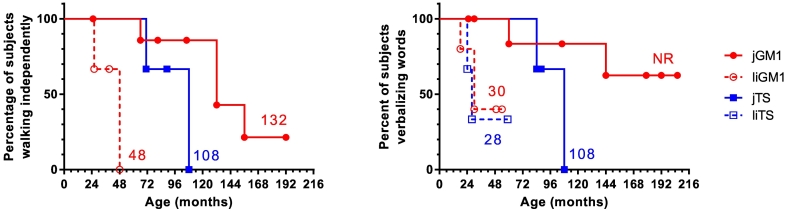
Kaplan-Meier analysis of the ability to walk independently and to verbalize words. Patients who never gained the ability to walk independently (*n* = 5) were not included in the analysis. Numbers indicate the median age (in months) at which ability was lost. jGM1, juvenile GM1-gangliosidosis; jTS, juvenile Tay-Sachs disease; liGM1, late-infantile GM1-gangliosidosis; liTS, late-infantile Tay-Sachs disease; NR, median not reached.

The most common initial symptoms noticed before diagnosis were gait abnormalities, difficulties with balance, frequent falls, and speech difficulties (Table 1). In subjects with liGM1, the ability to verbalize words began declining between 17 and 22 months of age in four of the five subjects and at 10 months in one subject (Supplementary Table 1). Once verbalization skills were observed to be declining, progression to complete inability to verbalize any words occurred after only 8 months in subject #9, and after 12 months in subject #11 (Supplementary Table 1). Subject #12 had learned many words before 18 months of age, but then began showing a decline in verbalization, similar to subjects #9, #10, and #11. Subject #12, however, was still able to say 2–3 words at age 4.1 years (49 months). Subjects with liTS showed a similar pattern of decline in verbalization, with subjects #17 and #19 having lost all word verbalization by age 24 months and 28 months, respectively, and subject #18 having learned up to 50 words by age 24 months, but having lost ability to say all but 6 words by age 59 months. The complete inability to verbalize any words occurred at a median age of 30 months for liGM1 and 28 months for liTS (Fig. 1).

In contrast, in subjects with the classic juvenile phenotypes, language and ambulation abilities started to decline later, typically between 5 and 7 years of age, and the rate of decline was more gradual than in subjects with the late-infantile phenotypes. Some subjects with jGM1 and jGM2 maintained these abilities after the age of 8 years. From the Kaplan-Meier analysis, subjects with jGM1 lost their abilities to walk independently and verbalize any words after the age of 11 years (median of 132 months and not reached, respectively). Subjects with jTS lost their ability to walk independently at 6–9 years and their ability to verbalize words at 7–9 years of age (median of 108 months for each). All the non-verbal subjects were still able to make vocalizations.

#### 3.3.2 Toilet training

Although toilet training was not a skill leading caregivers to seek a diagnosis, the caregivers expressed the importance of this skill towards quality of life issues for the subjects, and caregivers, including caregivers at the subjects' schools. Toilet training was documented to have been achieved in nearly all subjects with classic juvenile disease (6 of 8 subjects with jGM1 and all 3 subjects with jTS). The age of toilet training achieved ranged from 24 months to 36 months. All subjects with classic juvenile disease lost this skill (between age range of 5.5 years to 12 years), with the exception of 1 subject with jGM1 who was 16 years at age of last evaluation and was still able to alert caregiver of need to go to the toilet. In contrast, none of the subjects with liGM1 achieved toilet training. It is unknown if any subject with liTS achieved toilet training.

#### 3.3.3 Neurodevelopmental tests

Neurodevelopmental tests were administered to subjects whenever possible, and it was largely dependent on whether the subjects' health care plan paid for such testing. Twelve subjects (8 of 13 subjects with GM1-gangliosidosis and 4of 6 subjects with GM2-gangliosidosis) had at least one test administered. Eleven subjects had the Vineland test administered (the Vineland-II in all but one subject and the Vineland-III in the remaining one). Six subjects had the Bayley-III administered, whereas two subjects (both with jGM1) were higher functioning and had the KABC-II administered instead.

**Fig. 2:**
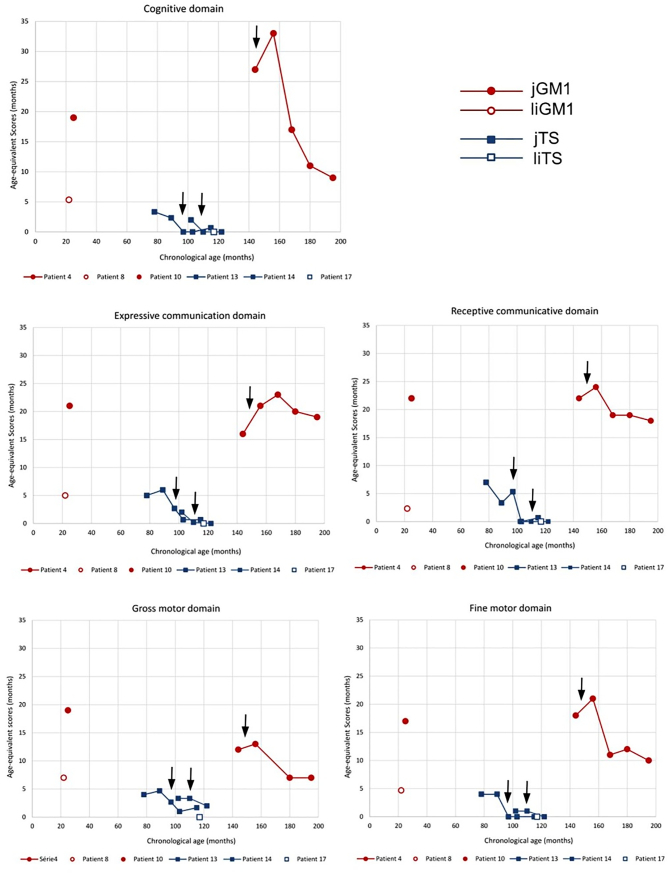
Bayley age-equivalent scores. Black arrows indicate when the patients received the full dose of miglustat.

Age-equivalent scores obtained with the Bayley-III showed that all subjects were developmentally impaired across the different domains of functioning (Fig. 2). Expressive and receptive communication scores as well as fine and gross motor scores were generally similar and declined similarly for a given subject. The performance of all three jGM2 subjects tested between the ages of 6.5 and 10.2 years were below the 8-month age-equivalent level, with only one subject exceeding the 5-months age-equivalent level for the language scales domain. One subject (#1) had KABC-II administered twice. Although the standard scores for the verbal knowledge and learning/memory domains were within the average range of functioning when they were 5 years of age, they were below average and impaired, respectively by the time they reached 6 years of age.

**Fig. 3:**
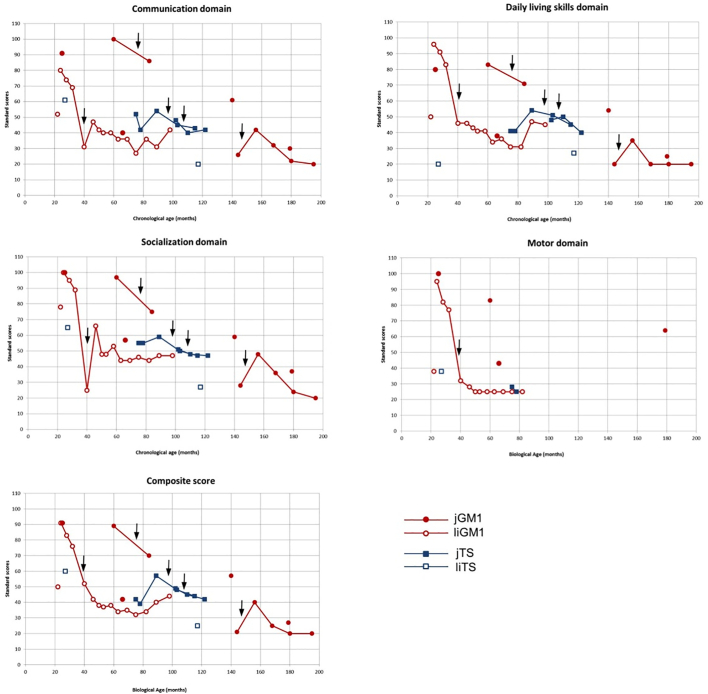
Vineland standard scores for each domain and the Adaptive Behavior Composite score. Black arrows indicate when the patients received the full dose of miglustat. All subjects but one had the Vineland-II administered. The last one had the Vineland-III administered.

Similarly, the results of the Vineland, which reflected the caregiver's perception of a subject's functioning when not in clinic, showed that most subjects (both classic juvenile and late-infantile disease) had a caregiver-reported level of functioning well below the normal range (i.e., standard scores of 85–115) (Fig. 3). Only three subjects, all 3 with classic jGM1 and under the age of 3 years, had any initial scores for the four subdomains within the average range. Importantly, these scores showed a decline sharply over time for the two subjects who had more than one test administered. All the subjects with jGM2 were endorsed by their caregivers to fall well below age-expectation across adaptive skills domains in their day-to-day environments.

#### 3.3.4 Seizure disorders

**Fig. 4:**
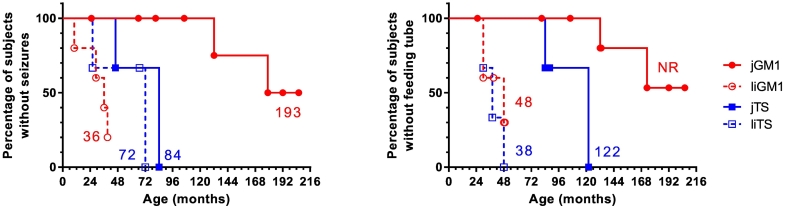
Kaplan-Meier analysis of onset of seizures and feeding tube placement. Numbers indicate the median age (in months) at seizure onset and feeding tube placement. jGM1, juvenile GM1-gangliosidosis; jTS, juvenile Tay-Sachs disease; liGM1, late-infantile GM1-gangliosidosis; liTS, late-infantile Tay-Sachs disease; NR, median not reached.

All three subjects with jTS had a seizure disorder (onset 46 months to 7 years) and two of the three subjects with liTS had documented seizures (onset 26 months and 6 years). Subject #18 with liTS had no documented seizures at the time of last evaluation (age 59 months). Three of 4 subjects with liGM1 had documented seizures (onset age 28.8 months to 40 months), and one subject had no known seizure history at the last evaluation (39 months). In contrast, only 3 of the 8 subjects in the jGM1 group had documented seizures, with the age of onset ranging from 3 to 11 years. The 5 subjects with jGM1 without documented seizures ranged in age from 5.5–17.2 years. The median age at seizure onset was 36 months for subjects with liGM1, 7 years for subjects with jGM2, and 16.1 years for subjects with jGM1 (Fig. 4). Although all the subjects with seizures received anti-seizure medications, most suffered multiple episodes each day. The presence of seizures was confirmed through documentation of seizures observed under medical observation and EEGs were performed to document absence of seizures.

#### 3.3.5 Other clinical symptoms

A cherry-red spot was observed in the macula of the eye in all three subjects with liTS and in 2 of 3 subjects with jTS, and faint haze in each macula in the third subject with jTS. In contrast, no cherry red spots were found in any of the subjects with GM1-gangliosidosis who were evaluated (6 of 8 subjects with jGM1 evaluated and 3 of 4 subjects with liGM1 evaluated).

Strabismus was a common finding in both GM1- and GM2-gangliosidosis subjects. In three subjects, strabismus was found earlier than the diagnosis of gangliosidosis.

Dysphagia, excessive oral and respiratory secretions, gastroesophageal reflux, and constipation were frequently reported across all groups. Hepatomegaly was reported only in two subjects (one with jGM1 and one with liGM1).

Kyphosis, scoliosis and hip dysplasia were common findings in jGM1 and liGM1, but were absent in subjects with jTS and liTS. Two subjects (one liGM1 and one liTS) experienced leg fractures between 5 and 8 years of age.

### 3.4 Medical interventions

#### 3.4.1 Feeding tubes

A feeding tube had been placed in the majority of subjects with liGM1, jTS, and liTS, due to dysphagia and subsequent risk of malnutrition and dehydration (Supplementary Table 3). In contrast, only 2 of 8 subjects with jGM1 had received a feeding tube. Placement was performed earlier in subjects with the late-infantile phenotypes (30 to 48 months in subjects with liTS and 48 months in subjects with liGM1) than in those with the juvenile phenotypes (6.8 to 10.2 years in subjects with jTS and 11.0 to 14.4 years in subjects with jGM1) (Fig. 4). The median age at placement was 3.2 years for liTS, 4.0 years for liGM1, and 10.2 years for jTS.

#### 3.4.2 High frequency chest wall oscillation therapy

High frequency chest wall oscillation therapy was initiated as palliative therapy to manage excessive respiratory secretions and mitigate respiratory infections in six subjects (one with jGM1, two with liGM1, one with jTS, and two with liTS).

#### 3.4.3 Miglustat therapy

Seven of the 19 subjects were on miglustat therapy combined with a ketogenic or low-carbohydrate diet (subjects #1, #2, #4, #9, #14, #15, #19). Miglustat was dosed based on BSA and according to dosing schedule used for children with Niemann Pick disease type C, per manufacturer label [21], Miglustat is a substrate reducing agent that inhibits glucosylceramide synthase in the glycosphingolipid pathway and thereby theoretically would be anticipated to reduce production of downstream products of that pathway, including GM1- and GM2-ganglioside [21,22].

Miglustat also inhibits disaccharidases in the gut, which leads to gastrointestinal symptoms of diarrhea, nausea, and abdominal pain if carbohydrates in the diet are not reduced [21]. The ketogenic diet combined with miglustat therapy helps to minimize risk of gastrointestinal side effects and may also improve seizure control. Arrows on the graphs of the neurodevelopmental testing results indicate age at which miglustat therapy was established (Fig. 2, Fig. 3). Some slight and transient improvements occurred in some of the subjects who were on miglustat therapy, as indicated by temporary increase in some of the neurodevelopmental test scores. No significant overall clinical change or improvements were observed, however, due to miglustat therapy, albeit the population was too small to make any conclusions.

### 3.5 Overall survival

**Fig. 5:**
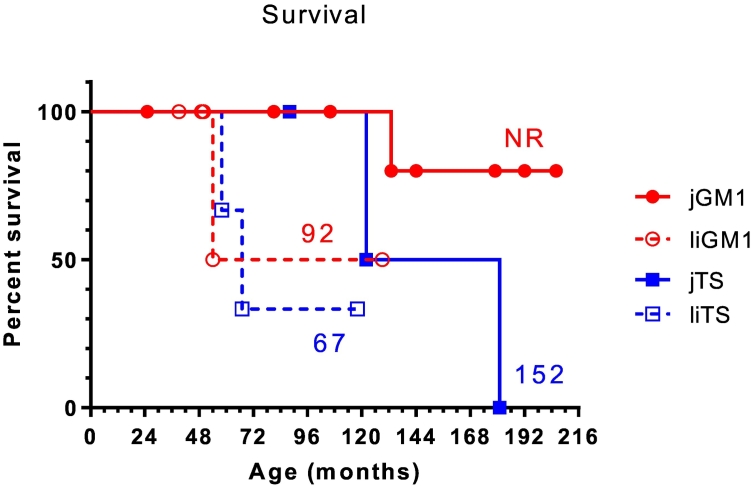
Kaplan-Meier analysis of overall survival. Numbers indicate the medians (in months). jGM1, juvenile GM1-gangliosidosis; jTS, juvenile Tay-Sachs disease; liGM1, late-infantile GM1-gangliosidosis; liTS, late-infantile Tay-Sachs disease; NR, median not reached.

Six subjects died during the study, 1 with jGM1, 2 with liGM1, 2 with jTS and 2 with liTS. Based on the Kaplan-Meier analysis the median overall survival was 12.6 years for jTS, 7.7 years for liGM1, and 5.6 years for liTS subjects (Fig. 5). The median overall survival was not reached for subjects with jGM1. The cause of death was unknown in the subject with jGM1 and respiratory failure associated with aspiration pneumonia in the other 5 subjects.

## 4 Discussion

This is the first natural history study to prospectively describe a timeline of clinical changes and to report systematic neurodevelopmental testing in the juvenile gangliosidoses. It is also the first natural history study to describe a late-infantile GM2-gangliosidosis phenotype, specifically, late-infantile Tay-Sachs disease. These findings are significant for clinical trial design. Moreover, the limited understanding, to date, of the patterns and ages of disease progression for the juvenile gangliosidoses has created barriers in clinical trial design for these conditions. Such knowledge deficits will also lead to barriers in future development of newborn screening programs for gangliosidoses.

The heterogeneity of the juvenile gangliosidoses is in sharp contrast to a more homogeneous phenotype that occurs in the infantile gangliosidoses. A timeline of clinical changes in the infantile forms of gangliosidoses has been previously described by Jarnes-Utz, et al. [5]. Initial symptoms of the infantile forms include profound hypotonia and developmental delay, followed by progressive neurological decline of motor skills, development of feeding difficulties, and onset of seizures. This pattern is markedly homogenous within the infantile GM1-gangliosidosis group and, likewise, within the infantile GM2-gangliosidosis group. In line with infantile phenotype homogeneity, there are also striking similarities between the infantile GM1- and GM2-gangliosidosis phenotypes in terms of the timeline for initiating various palliative care measures [5]. In contrast to the infantile forms, the clinical course of the juvenile phenotypes, for both GM1- and GM2-gangliosidosis, is more heterogeneous in the ages associated with of onset of various symptoms, and clinical features [1,4,7,23]. Defining the patterns of clinical disease progression of the juvenile form has been further complicated by the recognition of a subset of the juvenile phenotype of GM1-gangliosidosis, referred to as a late-infantile phenotype [9,11,13,14] and the few case reports of a late-infantile form of GM2-gangliosidosis [13].

### 4.1 Neurodevelopmental testing

Accurately evaluating the developmental abilities of children with a gangliosidosis condition is challenging due to a number of factors, most predominantly the severe and progressive neurological impairment and the challenges associated with quantifying a degenerative process in childhood. That said, standardized neurodevelopmental tests administered by experienced neuropsychologists are considered reliable and reproducible. If the measures are selected based on the level of functioning of the subjects estimated for the duration of the study, they would thus provide meaningful outcome measures for future trials evaluating treatments for GM1- and GM2-gangliosidoses, as suggested for other neurodegenerative diseases [24]. Regardless of the neurodevelopmental tests that will be used, they should be administered regularly, ideally at 6–12-month intervals, to allow proper longitudinal assessment of disease progression. This is particularly necessary for the Bayley-III, which is sensitive to the conditions and the child's status during the evaluation sessions (e.g., if they did not sleep well the previous night, if they are sick, if they have a seizure during the testing session, etc.) It is for this latter reason that use of a second, caregiver report measure (e.g., Vineland), is all the more important to gather information about the child's typical functioning and corroborate the data gathered with the neuropsychologist-administered measure (i.e. the Bayley-III). Despite some occasional variability in a child's performance on direct testing, the Bayley-III and Vineland tend to show skill gains and losses in parallel. Composite scores for the different domains of the Vineland generally mirror those obtained for their respective subdomains. However, some subdomains (e.g., Written in the Communication domain, Domestic and Community in the Daily Living Skills domain, and Coping in the Socialization domain) appear less useful than others (e.g., Receptive and Expressive in the Communication domain, and Interpersonal Relationships in the Socialization domain) as the most basic items in these former domains are beyond the capabilities of those children severely impacted by their gangliosidosis (data not shown).

### 4.2 Ambulation and verbalization skill decline led to diagnosis

This study highlights that for the juvenile gangliosidoses, the most common symptoms prompting the search for an inherited genetic disease diagnosis are development of difficulties with ambulation and changes in verbalization of words.

Importantly, most subjects, whether late-infantile or classic juvenile phenotype, were diagnosed before they had completely lost the ability to independently ambulate and verbalize words. The gradual loss of these abilities, occurring often after the diagnosis has been made, suggests stabilization of ambulatory and/or verbalization skills may be worth considering as a meaningful outcome measure for treatment trials for the juvenile gangliosidoses.

Ambulation and verbalization skill decline occurred most often in the 2nd year of life in the late-infantile subjects but not until the 3rd to 5th year of life in the classic juvenile phenotypes.

### 4.3 Toilet training

Achievement of toilet training is indicative of cognitive and motor development, as well as an activity of daily living associated with quality of life and impacting social and psychological well-being. Importantly, nearly all the subjects with classic juvenile gangliosidoses (both jGM1 and jGM2) achieved toilet training and achieved it within normal developmental ages for learning this skill, and all but one subject with jGM1 subsequently lost this skill. In contrast, none of the subjects with liGM1 were able to achieve toilet training.

### 4.4 Additional comparisons between juvenile GM1- and GM2-gangliosidosis

#### 4.4.1 Seizures

Seizure disorders were present in nearly all subjects with a diagnosis of liGM1, liTS and jTS, but less common in subjects with jGM1. (Supplementary Table 3). Notably, at the time of last evaluation, documented seizures were present in only 3 of the 8 subjects with jGM1, with onset ranging from age 36 months to 11 years. Moreover, 2 subjects with jGM1 were well into their adolescent years, with not known history of seizures.

#### 4.4.2 Cherry-red spots

Cherry-red spots were common in subjects with Tay-Sachs disease (present in 5 of 6 subjects), but absent in all 9 of the subjects with GM1-gangliosidosis who underwent evaluation by an ophthalmologist (6 with jGM1 evaluated and 3 with liGM1).

#### 4.4.3 Ophthalmologic findings

Strabismus was a common finding in both GM1- and GM2-gangliosidosis subjects, although the age of this finding was not known for all subjects. It is worth noting that in 3 subjects, strabismus was found much earlier than the diagnosis of a gangliosidosis condition, but did not lead the caregivers or healthcare providers to seek a possible genetic cause.

#### 4.4.4 Skeletal abnormalities

Skeletal abnormalities were common findings in subjects with juvenile GM1-gangliosidosis, with kyphosis and hip dysplasia being most prevalent.

These findings are consistent with previous work by Ferreira, et al. (2020) and Lang, et al. (2019), showing skeletal abnormalities as an important distinguishing feature of the GM1-gangliosidoses, present in both infantile and juvenile phenotypes [23,25].

#### 4.4.5 Feeding tube placement

At time of study analysis, a feeding tube had been placed in the majority of subjects with liGM1, jTS and liTS, but only 3 of 8 subjects with jGM1. Feeding tube placement was performed much earlier, before 4 years of age, in subjects with the late-infantile phenotypes, versus those with the juvenile phenotypes (placed between 6.8 and 10.2 years in jTS and 11–14.4 years in jGM1).

## 5 Conclusion

This is the first natural history study to prospectively describe a timeline of clinical changes in the juvenile gangliosidoses, to compare and contrast the timelines for the different phenotypes, and to report serial neurodevelopmental testing in the juvenile gangliosidoses. This is also the first study to formally propose a late-infantile GM2-gangliosidosis phenotype (i.e., late-infantile Tay-Sachs disease).

The consistency in the development of difficulties with ambulation and verbalization, as well as partial retention of these skills at time of diagnosis, suggests changes in ambulation and verbalization skills may play key roles as outcome measures in future clinical trials.

Delay to diagnosis of juvenile gangliosidosis conditions will create challenges for recruitment of subjects for clinical trials and compromise efficacy of promising therapies. Thus early diagnosis will be critical for optimizing advances in therapies for these conditions.

As strabismus and abnormal eye movements may be early signs of a juvenile gangliosidosis condition, more in-depth follow-up of children with these ophthalmologic abnormalities may lead to earlier diagnosis followed by earlier access to therapies for the gangliosidoses.

Finally, this study demonstrates the importance and feasibility of tracking with neuropsychological measures the neurodevelopmental progression of individuals with the juvenile gangliosidoses as well as treatment-related effects. The use of both direct evaluation of the patients by an examiner skilled in assessing children and infants with significant neurological impacts as well as caregiver rating forms (i.e. the Vineland) is recommended. This combination allows one to gather more objective data on the neurodevelopmental skills that can be demonstrated on command at a single time point as well as data on day-to-day functioning that may not always be able to be elicited in the testing environment. While some individuals with the juvenile forms of the gangliosidoses are able to complete neuropsychological measures designed for their age (e.g. the KABC-II), the individuals with the late-infantile forms as well as those with the more progressed juvenile forms require the use of the Bayley, a measure standardized for infants and toddlers. However, tracking skill gains/losses with raw scores and/or age-equivalents is still useful until the latest stages of neurodegeneration. Similarly, the Vineland, while useful to give in its entirety, some scales are more beneficial than others for individuals with the most progressed conditions. Given the difficulty quantifying skills for those individuals with the most significant gangliosidoses-related impacts, it once again highlights the importance for clinical trial endpoints of early identification of these diagnoses, prior to significant skill losses.

## Funding

This work was supported by the 10.13039/100000002National Institutes of Health through the Lysosomal Disease Network (U54NS065768). The Lysosomal Disease Network (U54NS065768) is a part of the National Institutes of Health (NIH) Rare Diseases Clinical Research Network (RDCRN), supported through collaboration between the NIH Office of Rare Diseases Research (ORDR) at the National Center for Advancing Translational Science (NCATS), the National Institute of Neurological Disorders and Stroke (NINDS), and the National Institute of Diabetes and Digestive and Kidney Diseases (NIDDK).

## Role of the funding source

The funders had no role in study design; in the collection, analysis and interpretation of data; in the writing of the report; and in the decision to submit the article for publication. The content is solely the responsibility of the authors and does not necessarily represent the official views of the National Institutes of Health.

## Declaration of Competing Interest

No conflicts of interest to declare.

## Appendix A Supplementary data

Supplementary material. Image 1
