# Supplementary material for: The juvenile gangliosidoses: A timeline of clinical change
Source: Mol Genet Metab Rep. 2020 Nov 14;25:100676. doi: 10.1016/j.ymgmr.2020.100676 (PMC7674119; doi:10.1016/j.ymgmr.2020.100676)
Supplement: Supplementary file 1 — Supplementary material. [file mmc1.docx]

# Supplementary material

## Supplementary Table 1. Developmental milestones gained and lost

| **Subject/**  **Diagnosis/**  **Gender/**  **(Age at last evaluation)** | **Were first-year developmental milestones met*** | **Changes in ambulation (ages of events)** | | | **Changes in vocabulary and verbal communications** | | | **Changes in learning skills and activities at home and/or school.** | **Toilet training** |
| --- | --- | --- | --- | --- | --- | --- | --- | --- | --- |
|  |  | **Age at which subject learned to walk independently** | **Onset of gait abnormalities or difficulties with balance** | **Subsequent changes in ambulation** | **Age at which subject learned first words** | **Number of words spoken** | **Age at which speaking difficulties first noted.**  **Subsequent speaking changes.** |  |  |
| #1  jGM1  F  (12 years) | Yes. | 12 months | 5.5 years: falling down frequently while walking, gait abnormalities, and falling off chair at school. | Non-ambulatory at 11 years. | 8 months | Hundreds of words; spoke in full sentences. | Dysphasia noted at 5.5 years.  Loss of ability to say any words at 12 years. | Began school at 48 months. Continues to attend school for 2 h per day. Lost ability to read and write at 9 years, after which time subject was schooled in special needs classroom full-time. Could do math through 11 years, then lost this ability. | By 36 months; lost this ability at 6 years. |
| #2  jGM1  F  (16 years) | Yes. | 13 months | 30 months: falling down frequently at pre-school. | Continues to walk independently at 16 years; uses wheelchair for longer distances such as airport or visiting museum. | 12 months | More than 500 words; has maintained ability to speak these words. | Dysphasia noted at 30 months.  Continues to speak at 16 years, albeit with impediments. | Attends school. Learned to read and write at 5 years. Diagnosed with inattention at 9 years. At 13 years was doing math and reading at 9-year old level. Can type words on iPad and write words adaptively at 16 years. | By 24–36 months; still toilet trained at 16 years, but asks for assistance if needed. |
| #3  jGM1  F  (14.9 years) | Yes. | 12 months | 5 years: difficulties with balance during walking. | No longer walking at 13 years, but could crawl. | 12 months | Numerous words; spoke in full sentences. | Began losing words at 48 months.    By 14 years could only say “mom”. | Began school at 5 years. Unknown if continued in school. Developmental delay and autism diagnosis at 60 months (7 years before jGM1 diagnosis). Showed indication of understanding of simple commands and “yes” and “no” at 14.9 years. | Toilet trained, age unknown. Lost this ability, age unknown. |
| #4  jGM1  M  (17.2 years) | Yes. | 12 months | 36 months: falling down frequently while walking. | Gait abnormalities, difficulty with stairs and getting into and out of cars noted at 8 years. Unable to walk independently at 10 years. At 17.2 years, can walk with walker. | 9 months  (“mama” and “dada”) | 150 words; used 3–4 word phrases by 3 years; spoke in full sentences. | Developmental speech delay at 24 months, which improved after ear tubes placed at 30 months.  Started losing words at 5 years.  By 15 years, could only speak in one-word statements but could say 30 different words. | Attended preschool at 48 months and regular kindergarten at 5 years. Diagnosed with educational autism at 5 years old. Transferred from regular kindergarten to special education schooling at 5 years. Never learned to read or write, but can recognize letters of alphabet. Learned to draw basic shapes, count to 100, and recognize numbers. | By 36 months; lost this ability at 12 years. |
| #5  jGM1  F  (6.8 years) | Yes. | 13 months | 18 months: gait abnormalities noted, balance difficulties while walking. | Still walking independently at 6.8 years, albeit with abnormal gait. | 13 months | Unknown. | Stuttering and speech delay first noted at 30 months.  Subsequent changes unknown. | Unknown. | Unknown. |
| #6  jGM1  M  (8.8 years) | Yes.  Lifted head while on tummy at 2−3 months,  held head up independently by 4 months,  sat independently (without support) and crawled at 6  months. | 9 months | 54 months: frequent falls and clumsiness. | Continued to ambulate independently at last evaluation (8.8 years). | 12 months | “Many words.” Spoke in full sentences by 24 months. Held conversations with others. | Began having difficulties with speech, a new onset of stuttering, and began losing words at 54 months.  By 8 years, could only say 4 words (“mommy”, ”brother”, grandpa”, “pee”). | By 36 months, could count to 20 and say half of ABCs. Bathed himself at 24 months, but lost this ability by 8 years. Dressed himself at 36 months, but began having difficulty at 54 months. Diagnosed with attention deficit, age unknown.. | Toile trained, age unknown. By 24 months; developed urinary incontinence at 8.5 years, and would say “pee” to mother when incontinent. |
| #7  jGM1  M  (5.5 years) | Yes. Crawled at 7 months. | 10 months | 48 months: gait abnormalities and frequent falls. | Diagnosed with ataxic cerebral palsy at 5 years. At 5.5 years, unable to stand or walk independently (onset unknown), could still stand unassisted for few seconds and walk with assistance. | 12 months | 5 words. | Began losing words after 36 months.  Could no longer speak any words by 5 years. | Attended kindergarten through special education program. “Severe delays in reading, mathematics and written language”. At 5.5 years, reliant on caregivers for all daily living skills.. | Achievement of toilet training (age unknown; not toilet trained at 5.5 years. |
| #8  jGM1  M  (25 months) | Yes. | 10 months | Still able to walk, run, and climb stairs, throw a ball, climb on adult-sized furniture. No gait abnormalities noted at neurology evaluation. | No regression. No gait abnormalities noted at neurology evaluation | 12 months | Spoke about 20 words, but demonstrated understanding of many more words than he could speak. | At last evaluation, subject had speech delays but was continuing to learn new words. Could talk to a familiar person on the telephone. | Able to turn book pages one-by-one. Able to participate in dressing routines, feed himself with utensils, and wipe up spills. | Not achieved. |
| #9  liGM1  M  (129 months) | Yes.  Rolled over without assistance first week of life, held head up independently at 3 months, crawled at 4 months, furniture surfed at 7 months. | 9 months | 20 months | Lost ability to walk independently at 26 months. Was able to ambulate slowly in walker, with assistance, until bilateral hip dislocation at 8 years. Was able to kick on command during therapies until 10 years. | 10 months | Said “mamma”, “hello”, “hi”, “light”. Pointed at objects and said, “that” and “what”.  Never learned to string 2 or more words together. | Began losing words at 22 months.  Vocabulary became gradually smaller. Stopped saying words at 30 months. Continued to communicate by pointing at things he wanted. Brought things he wanted to parents until he lost ability to walk independently at 26 months | Never learned to read, write or do math. | Not achieved. |
| #10  liGM1  M  (42 months) | Yes. | Never learned to walk independently; furniture surfed at 12 months. | 17 months: balancing difficulties and falling down frequently. | Lost ability to furniture surf by 18 months. | Within 1^st^ year; age unknown | Unknown. | Began having difficulties with speaking at 17 months. | Unknown. | Unknown. |
| #11  liGM1  M  (50 months) | Yes. | 12 months | 18 months: balancing difficulties while walking. | Non-ambulatory by 48 months.  Can still sit unaided at 50 months. | Unknown | Unknown. | Began losing words at 18 months. Lost all words by 30 months | Unknown. | Unknown. |
| #12  liGM1  F  (49 months) | Yes. | Learned to crawl during 1^st^ year of life. Never learned to walk independently. | Never learned to walk. | Lost ability to crawl during 3^rd^ year of life. | Within 1^st^ year; age unknown | Spoke numerous words. | Diagnosed with speech developmental delays and global developmental delay at 18 months. Began losing words after 18 months.  Could say only 2−3 words at last evaluation, and seldom tried to speak. | Unknown. | Not achieved. |
| #13  liGM1  F  (39 months) | Yes. Crawled at 6 months, surfed furniture at 11 months. | 13 months | 18 months: balancing difficulties while walking. | Continues to walk independently, with abnormal gait at 39 months | 8−10 months | Two words. | No new words after 10 months.  Lost ability to say words at 18 months | Unknown. | Not achieved. |
| #14  jTS  F  (9.6 years) | Yes. | 12 months | 36 months: balancing difficulties while walking. | Lost ability to walk independently at 5.9 years | Within 1^st^ year of life; age unknown | Spoke numerous words. | Developed stuttering at 36 months and showed progression of speaking difficulties with loss of words at 5 years.  Lost all ability to speak words by 84 months (7 years) | Unknown. | Toilet trained, age unknown; lost this ability at 5.7 years |
| #15  jTS  F  (10.2years) | Yes. | Around 12 months | 5.5 years: balancing difficulties while walking. | Became increasingly cautious about jumping, stair climbing, running at 5.5 years. Began falling at notably increased frequency at 7 years.  Balance difficulties continued to worsen, non-ambulatory by 9 years. | Within 1^st^ year of life; ; age unknown | Had large vocabulary and spoke in full sentences by 36 months | Began stuttering, having difficulty finding words, and began losing words at 5 years. Continued to progressively lose ability to say words.  By 7 years, could still occasionally say words and short phrases, but could not speak in full sentences.  Lost ability to speak any words by 9 years. | Unknown. | Toilet trained, age unknown; lost this ability, age unknown. |
| #16  jTS  F  (7.3 years) | Yes.  Held head up independently at 5 months, sat independently at 6−7 months, crawled at 7 months, furniture surfed at 7–8 months. | 14 months | 36 months years: falling frequently. | Still walking independently at 7.3 years. | Within 1^st^ year of life; age unknown | Learned many words. Was speaking well at 24 months and gained new words and improved speaking skills until 6 years. | Began stuttering at 42 months. | Learned alphabet. Did not learn to read. At age of last evaluation, continued to attend regular school, could count to 20, does math in school but does not have passing grades in math. | At 36 months; lost this ability at 7 years. |
| #17  liTS  F  (2.2 years) | Yes. Sat independently by 7−8 months, furniture surfed by 10 months. | Never learned to walk independently. | 14 months: furniture surfing became difficult. | Could no longer furniture surf or sit independently | Unknown | Unknown. | Could not speak any words. | Unknown. | Unknown. |
| #18  liTS  F  (4.9 years) | Yes. | Learned to furniture surf around 12 months. Never learned to walk independently. | 14 months: furniture surfing became difficult. | Between 12–18 months, could take steps if someone was supporting her arms and could pull herself up to standing position. Lost these abilities between 24–30 months. | Unknown | Spoke 30−50 words by 24 months. | Began losing words at 30 months.  By 59 months, could only say 6 words. | Unknown. | Unknown. |
| #19  liTS  F  (9.8 years) | Yes. Held head independently and lifted head while on tummy by 3 months, rolled over at 3–4 months, sat independently and crawled at 6 months. | Learned to furniture surf around 10 months. Never learned to walk independently. | 16 months: furniture surfing became difficult. |  | 8–9 months | 10–20 words. | Started losing words at 16–17 months.  Non-verbal at 28 months. | In special education program in the 3^rd^ grade. Seldom opens eyes, but can smile in response to voices of parents and teachers. | Unknown. |

*First-year developmental milestones: see **Supplementary Table 2** describing first-year milestones (gross motor, fine motor, language, cognitive, and social)

## Supplementary Table 2. First-year developmental milestones

| **Age** | **Gross Motor** | **Fine Motor** | **Language/Cognitive** | **Social** |
| --- | --- | --- | --- | --- |
| **1 month** | Moves head from side to side when on stomach | Strong grip | Stares at hands and fingers | Tracks movement with eyes |
| **2 months** | Holds head and neck up briefly while on tummy | Opens and closes hands | Begins to play with fingers | Smiles responsively |
| **3 months** | Reaches and grabs at objects | Grips objects in hands | Coos | Imitates you when you stick out your tongue |
| **4 months** | Pushes up on arms when lying on tummy | Grabs objects – and gets them! | Laughs out loud | Enjoys play and may cry when playing stops |
| **5 months** | Begins to roll over in one or the other direction | Is learning to transfer objects from one hand to the other | Blows “raspberries” (spit bubbles) | Reaches for mommy or daddy and cries if they’re out of sight |
| **6 months** | Rolls over both ways | Uses hands to “rake” small objects | Babbles | Recognizes familiar faces – caregivers and friends as well as family |
| **7 months** | Moves around – is starting to crawl, scoot, or “army crawl” | Is learning to use thumb and fingers | Babbles in a more complex way | Responds to other people’s expressions of emotion |
| **8 months** | Sits well without support | Begins to clap hands | Responds to familiar words, looks when you say his name | Plays interactive games like peekaboo |
| **9 months** | May try to climb/crawl up stairs | Uses the pincer grasp | Learns object permanence – that something exists even if he can’t see it | Is at the height of stranger anxiety |
| **10 months** | Pulls up to stand | Stacks and sorts toys | Waves bye-bye and/or lifts up arms to communicate “up” | Learns to understand cause and effect (“I cry, Mommy comes”) |
| **11 months** | Cruises, using furniture | Turns pages while you read | Says “mama” or “dada” for either parent | Uses mealtime games (dropping spoon, pushing food away) to test your reaction; expresses food preferences |
| **12 months** | Stands unaided and may take first steps | Helps while getting dressed (pushes hands into sleeves) | Says an average of 2−3 words (often “mama” and “dada”) | Plays imitative games such as pretending to use the phone |

Adapted from <https://www.webmd.com/>

## Supplementary Table 3. Clinical events and findings (age of onset or finding)

| **Subject**  **Diagnosis**  **Gender**  **(Age of last evaluation)** | **Ophthalmologic findings** | | **Hearing impairment** | **Seizure onset** | **Sleeping difficulties** | **Cardiology findings** | **Gastrointestinal findings/events** | | | | **Hepato-megaly** | **Urinary Retention** | **Respiratory findings/events** | | | **Skeletal morphologic abnormalities and Orthopedic findings/events** |
| --- | --- | --- | --- | --- | --- | --- | --- | --- | --- | --- | --- | --- | --- | --- | --- | --- |
|  | **Cherry red spot** | **Vision abnormalities/**  **impairment/ eye movement abnormalities** |  |  |  |  | **Dysphagia noted** | **Feeding tube placed** | **Gastro-esophageal reflux disease** | **Chronic constipation** |  |  | **Excessive salivation** | **Pneumonia first experienced** | **Receiving high frequency chest wall oscillation therapy** |  |
| #1  jGM1  F  (12 years) | Absent | Corneal clouding and astigmatism; age of findings unknown. | No | 11 years | Onset at 10 years; managed with melatonin. | No abnormal findings on echo and electrocardiogram done at 6.6 years. | 11 years | 11 years | Absent | Absent | Absent | No | Absent | No history of pneumonia. | No | None noted. |
| #2  jGM1  F  (16 years) | Absent | Glasses pre-scribed at 5 years; no retinal or optic nerve disease found at this time. | No | No seizure history at age of last evaluation (16 years). | Normal sleep study at 13.3 years; parents report no sleeping difficulties observed at home at 16 years. | None (last evaluated at 13.7 years). | 13.3 years | None; continues to eat by mouth at 192 months of age;  often takes 2 h to eat meal. | Absent | Yes, onset unknown. | Absent | No | Onset at 12 years. | No history of pneumonia. | No | Vertebral breaking (MRI, 6 years); endplate abnormalities (MRI, 8 years).  Osteoporosis at 12.3 years months. |
| #3  jGM1  F  (14.9 years) | Absent | Some corneal clouding; age of finding unknown.  Slight near-sightedness diagnosed at 14 years. | Diagnosed with auditory processing disorder; age unknown. | Seizure disorder, onset unknown. | Obstructive sleep apnea diagnosed at 14.8 years;  C-PAP prescribed. | Very mild thickening of heart valves, the mitral > aortic at 14.9 years. | Unknown | 14.4 years | Not noted. | Yes, onset unknown. | Absent | Yes, onset unknown. | Noted at 15.25 years, onset unknown; suction and medication therapy prescribed. | No history of pneumonia. | 16 years | Hip dysplasia, kyphosis. |
| #4  J-GM1  M  (17.2 years) | Absent | Stigmatism, onset unknown. | No | No seizure history. | Yes, onset unknown; managed well with melatonin started at 9 years. | Abnormal findings on echo and EKG done at 13.3 years. | Absent | None; continues to eat by mouth. | Absent | Yes, onset after starting low-carbohydrate diet at 12.1 years | Absent | Yes, onset at 12 years. | Absent | No history of pneumonia. | No | Kyphosis diagnosed at 8 years; surgery to correct spine curvature at 14.8 years; rib oaring/flaring identified at 16 years. |
| #5  jGM1  F  (6.8 years) | Unknown | Strabismus at 30 months. At 4 years, extra-ocular eye movements were noted to be full but with “saccadic undershoot”. | Unknown | No history of seizures. | Unknown | Not evaluated. | Not evaluated. | None; continues to eat by mouth. | Absent | Absent | Present; liver “moderately enlarged”; age of initial finding unknown. | Yes, onset unknown. | Absent at age of last evaluation (6.8 years). | No history of pneumonia. | No | Kyphosis present, age of finding unknown;  bilateral hip dysplasia found at 49 months. |
| #6  jGM1  M  (8.8 years) | Unknown | Unknown | Unknown | No history of seizures. | Yes, onset unknown; managed with clonidine at bedtime. | Cardiology evaluation not done. | Not evaluated. | None; continues to eat by mouth. | Absent | Yes, onset unknown. | Absent | Yes, onset unknown. | Absent | No history of pneumonia. | No | Slight curvature of spine found at 8 years. |
| #7  jGM1  M  (5.5 years) | Absent | Amblyopia, esotropia, hyperopia with astigmatism at 24 months. Visual tracking highly variable at 5.5 years. | Normal hearing evaluated at 5 years. | No seizure history. | None | Unknown | 5 years | None; continues to eat by mouth. | Unknown | Unknown | Absent | Absent | Absent | No history of pneumonia. | No | Abnormal vertebral body height and shape found on spine MRI at 5 years. |
| #8  jGM1  M  (2.1 years) | Absent | Strabismus at 24 months. | None | No seizure history. | None | Unknown | Absent | None; continues to eat by mouth | Unknown | Unknown | Absent | Absent | Absent | No history of pneumonia. | No | Unknown |
| #9  liGM1  M  (10.8 years) | Absent | Strabismus during 1^st^ year of life which resolved at 4.5 years. | Moderate to severe hearing loss due to GJB2 mutation. | 36 months | Yes, onset at 5 years, in which subject would not sleep for 2 to 3 nights in a row. Insomnia stopped at 8 years subsequent to beginning CBD oil.  Obstructive sleep apnea (uses biPAP during sleep). | Unknown | Unknown | 30 months | Unknown | Yes, began at 42 months after starting ketogenic diet. | Noted by pediatrician at 20 months. | Yes, onset at 7 years. | 6 years | Hospitalized for pneumonia at 4.3 years, 4.9 years (RSV), 6.1 years (RSV), 10.2 years, and 10.3 years. Suddenly stopped breathing for <1 min at 6.5 years while at school. | Started at 6.9 years, along with hand percussion pulmonary physiotherapy. | Mild kyphosis noted at 24 months. Compound fracture below knee occurred during seizure at 5 years. Osteoporosis diagnosed at 6 years. Bilateral hip dislocation at 8 years. |
| #10  liGM1  M  (3.5 year)s | Absent at 20 months. | Astigmatism found at 21 months; bilateral photoreceptor dysfunction found at 23 months; difficulty tracking noted at 28 months; prescription glasses at 36 months. | None | 29 months | Unknown | At 22 months, minimal mitral and aortic valve thickening, with no evidence of stenosis or aortic insufficiency. Normal ventricular contractility. | 30 months | 30 months | 30 months | Yes, onset 7 months. | Absent | Absent | Yes, onset around 30 months | 3 RSV pneumonia infections between 24–36 months. | Started at 24 months. | Kyphosis and bilateral hip dysplasia identified at 18 months. |
| #11  liGM1  M  (50 months) | Absent | Absent | None | 39 months | Unknown | Unknown | 48 months | G-tube placed at 48 months | Unknown | Unknown | Absent | Unknown | Unknown | Unknown | Unknown | Unknown |
| #12  liGM1  F  (49 months) | Unknown | Normal vision evaluation at 28 months. | Normal hearing evaluation at 19 months. | 40 months (3.33 years) | Unknown | Unknown | Unknown | No; continues to eat by mouth. | Unknown | Unknown | Unknown | Unknown | Absent | No history of pneumonia. | Unknown | Mild kyphosis noted at 49 months |
| #13  liGM1  F  (39 months) | Absent at 33 months. | Left eye esotropia and strabismus identified at 26 months. | Normal hearing evaluation at 27 months. | None | None | Normal heart echo at 22 months. | Present, onset unknown. | No; continues to eat by mouth. | Absent | Absent | Absent | Absent | Absent | No history of pneumonia. | No | Kyphosis (diagnosed at 27 months).  Gibbus at L2-L3 noted at 3 months. |
| #14  jTS  F  (9.6 years) | Mild  cherry red spots noted at 8.1 years | Intermittent nystagmus noted at 8.1 years | Mild hearing loss noted at 7.5 years. | Isolated seizure at 46 months.  Onset of chronic, recurrent seizure disorder at 7 years | Restless arms and legs during sleep, onset unknown. | Normal cardiac echo at 9.5 years. | During 6^th^ year of life | Feeding tube placed at 7 years | Unknown | Yes, onset unknown. | Absent | Unknown | Yes, onset unknown. | Hospitalized for pneumonia at 10.2 years during which time subject died due to respiratory failure. | Unknown | None noted. |
| #15  jTS  F  (10.1  years) | No; faint haze in each macula noted at 9 years, without discernable cherry red spot. | Hyperopia noted at 9 years. | None noted. | 7 years | Unknown | Unknown | Present, onset unknown. | 10.2 years | 10.2 years | Yes, onset unknown. | Absent | Unknown | Yes, onset unknown | Six hospitalizations for aspiration pneumonia between 10–15 years. Died secondary to respiratory failure during hospitalization for pneumonia at 15 years. | Yes, started at 11 years. | None noted. |
| #16  jTS  F  (7.3 years) | Bilateral cherry red spots found at 7.3 years | Stigmatism diagnosed and glasses prescribed at 6 years. | Normal hearing. | 7 years, spiking brain waves on EEG during sleep. | Unknown | Cardiology evaluation at 5 years showed no abnormalities. | Swallowing difficulties since 5 years. | Continues to eat by mouth. | Absent | Chronic constipation since 3 years. | Absent | Absent | Absent | No history of recurrent respiratory illnesses. | No | Not evaluated. |
| #17  liTS  F  (2.2 years) | Bilateral cherry red spots found at 26 months. | Optic atrophy, nystagmus. | None noted. | 26 months | Unknown | Unknown | 26 months | Feeding tube placed 30 months | Unknown | Yes, noted at 26 months. | Absent | Yes, noted at 26 months. | None noted. | 3 pneumonia infections at 33 months, 38 months and 40 months. | Yes, started at 29 months (2.4 years). | None noted. |
| #18  liTS  F  (4.9 years) | Cherry red spots found at 36 months. | Amblyopia found at 36 months. | Unknown | Absent | Unknown | Unknown | Present, onset unknown. | Feeding tube placed at 48 months. | Yes, onset unknown. | Yes, noted shortly after birth. | Absent | Absent | Yes, onset unknown. | None noted. | None noted. | None noted. |
| #19  liTS  F  (9.8 years) | Bilateral optic nerve pallor (faint cherry red spots) noted at 20 months. | Abnormal eye movements at 30 months resolved by 5 years. Eye tracking became markedly diminished at 4–5 years; tracking completely stopped by 6 years. | Unknown | 6 years | Unknown | Unknown | Unknown | 38 months | Yes, onset 30 months.  Hiatal hernia at 7 years. | Yes, onset unknown. | Absent | Yes, onset unknown. | Yes, onset unknown. | Hospitalized for pneumonia 1–2 times yearly, beginning 5 years.  Tracheostomy placed at 7.3 years). | Started at 4 years. | 3 femur fractures found and osteopenia diagnosed at 6 years. |

biPAP, bi-level positive airway pressure.
